# Uterine rupture in pregnancy subsequent to hysteroscopic surgery: A case series

**DOI:** 10.4274/tjod.77642

**Published:** 2017-12-30

**Authors:** Şahin Zeteroğlu, Melisa Aslan, Bertan Akar, Rukiye Ada Bender, Alper Başbuğ, Eray Çalışkan

**Affiliations:** 1 Bursa Acıbadem Hospital, Clinic of Obstetrics and Gynecology, Bursa, Turkey; 2 Acıbadem University, Vocaitonal High School, İstanbul, Turkey; 3 Bahçeşehir University Faculty of Medicine, İstanbul, Turkey; 4 İstinye University Faculty of Medicine, Department of Obstetrics and Gynecology, İstanbul, Turkey; 5 Düzce University Faculty of Medicine, Department of Obstetrics and Gynecology, Düzce, Turkey; 6 Bahçeşehir University Faculty of Medicine, Department of Obstetrics and Gynecology, İstanbul, Turkey

**Keywords:** Hysteroscopy, uterine rupture, pregnancy

## Abstract

Uterine rupture during pregnancy is associated with high mortality and morbidity rates in both the fetus and the mother. Hysteroscopic surgeries such as myomectomy and septum resection are known risk factors for uterine rupture in pregnancy following the operation. We present four infertile patients who were admitted to Kocaeli Medical Park Hospital between February 2014 and November 2016. Three of the patients underwent hysteroscopic septum resection without complication and one had hysteroscopic myomectomy and a 7-8 mm sized rupture was detected. All of the patients became pregnant in less than a year after the operations. The first three patients had uterine rupture at 22^nd^, 38^th^, and 10^th^ week, which is the earliest rupture in the literature. The last patient had an uneventful pregnancy and the rupture was observed during cesarean section. A short interval between hysteroscopy and pregnancy may increase the risk of rupture. It may be possible to become pregnant despite rupture and not have any problems during the entire pregnancy.

## INTRODUCTION

Hysteroscopy is a routinely applied procedure for cervical canal and uterine cavity visualization. It is preferred in diagnosis, sample taking, and also in intrauterine surgeries. In septum resection, synechiolysis, myomectomy of submucosal myomas, and polypectomy, hysteroscopy is accepted as the standard treatment. Although diagnostic hysteroscopy is significantly safe compared with surgical hysteroscopy, there is a rate of 0.95% complications in surgical applications^(1)^. The complications can be listed in the order of frequency as perforation (instrumental, hysteroscopic) and fluid overload. The rate of complications in different procedures of hysteroscopy differ. Although adhesiolysis has significantly higher incidence of complications, it is followed by endometrial resection, myomectomy, and polypectomy, which are not significantly different from each other^(1,2)^.

Uterine rupture is both intra-operative and one of the late complications of hysteroscopy. Although it is a rare complication, it can be catastrophic if not managed properly and may result in fetal and maternal death. Uterine perforation and metroplasty (both abdominal and hysteroscopic) are risk factors of uterine rupture in subsequent pregnancies^(3,4)^. Physiologic distention of the uterus during pregnancy may increase the risk of rupture in scarred uteruses.

The purpose of this study was to present four cases of uterine rupture during pregnancy following a hysteroscopic surgery in 4 infertile patients who were admitted to Kocaeli Medical Park Hospital between February 2014 and November 2016. Approval was obtained from the local ethics committee to perform the study. Written informed consent was obtained from all participants.

## CASE REPORTS

### Case 1

A 24-year-old nulligravida, nulliparous woman presented with four years of primary infertility. The investigations of hysterosalpingograhy and 3D ultrasonography (USG) showed uterine septum and later the septum was resected hysteroscopically. During the 6 months of follow-up, the patient could not become pregnant. Despite gonadotropin application for two cycles and insemination because of astenospermia, the infertility of the patient continued. The patient was then treated with short-protocol in vitro fertilization (IVF) for one cycle. She had an uneventful pregnancy until she was admitted to hospital with abdominal pain at the 38th week. Despite reactive external fetal tocography without uterine contractions, cesarean section was performed because the patient’s pain persisted. A rupture with disruption of all layers measuring 4 cm was observed in the uterine fundus.

There was no accompanying intra-abdominal bleeding, fetus or amniotic sac protrusion. By lower segment transverse incision, a 3150 g male infant with Apgar score of 9-10 was delivered by its foot. The placenta was completely exteriorized from the uterus. Uterine rupture was repaired through a double-layer closure with 1.0 absorbable sutures. The mother and child were discharged two days later without any complications or problems and antibiotic therapy was given. No other problems were observed during the one-month follow-up of the patient.

### Case 2

A 39-year-old woman, gravida 6, abortus 5, with no living babies, underwent hysteroscopic surgery because of bicornuate uterus and complete septum. After her septum was resected hysteroscopically, in the laparoscopy a 1.5 cm sized indentation on the fundus was observed, and both tubes were open. No perforation was detected. Six months later, she became pregnant with twins through Klomen (Clomid) induction. The patient had no problems during her pregnancy until she presented to the clinic at the 10th week with periumbilical pain and bleeding. She underwent an emergency operation, and uterine rupture was detected on the fundus and closed. Six months later, the size of the rupture was measured as 5 mm using USG. She was followed up for three months and could not become pregnant with clomiphene citrate. After IVF treatment for one cycle, she had no results again. Six months after undergoing the Strassman metroplasty, controlled ovarian hyperstimulation IVF was started; follicle growth was not detected and the treatment was cancelled. She was followed up for natural cycles. In the 4th month, human chorionic gonadotropin was applied for one follicle, and she became pregnant. Her pregnancy was uneventful and a 3200 g infant was delivered by elective cesarean section at the 38^th^ week.

### Case 3

A 27-year-old woman, gravida 3 (1 ectopic, 1 chemical), parity 1, with no living babies, who had uterine septum with thickness of 2 cm, underwent hysteroscopic septum resection. No complications or uterine rupture were observed in the hysteroscopy. During the 6-month follow-up she could not become pregnant. IVF treatment with 3 embryo transfers was then applied. Her singleton pregnancy was uneventful until the 22^nd^ week, when she was admitted to hospital on foot with persistent right subcostal pain beginning in the night and vaginal spotting. On USG, amniotic membranes protruding from posterior side of the uterus were observed. She was underwent surgery immediately. In the operation, the uterine rupture measured 3 cm and was repaired using double-layer closure with 1.0 absorbable sutures. She was followed up for one year and no extra problems were observed.

### Case 4

**Figure 1:**
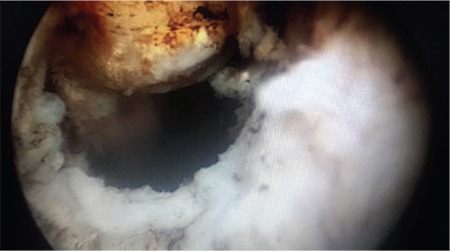
Perforation of the uterus during hysteroscopic myomectomy

**Figure 2:**
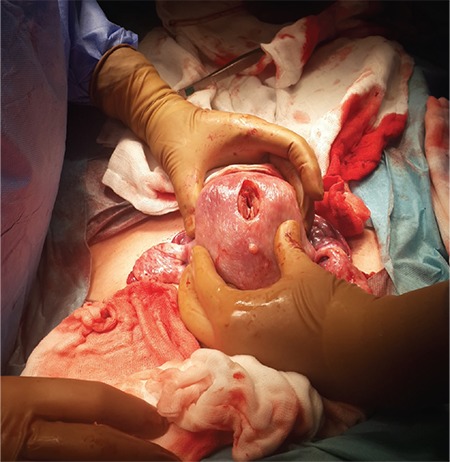
Full thickness, unhealed or scar dehiscence of previous perforation area noticed during elective cesarean section after the baby is delivered

**Figure 3:**
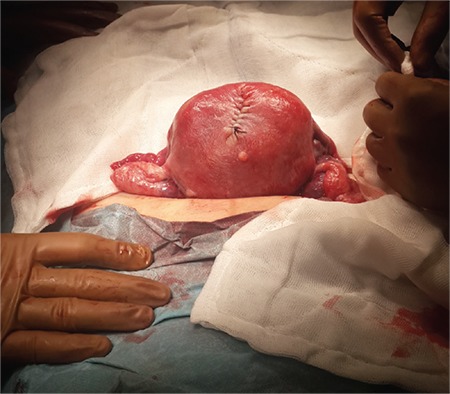
Uterine repair of the defect with two layer sutures

A 32-year-old nulliparous and nulligravida woman admitted to our clinic after reporting not being able to become pregnant during 2 years of marriage (December, 2014). She had regular but excessive bleeding. A type 3 23x18 mm submucous myoma was detected in the fundal region using transvaginal USG. During the hysteroscopic myomectomy, a 7-8 mm sized rupture near the fundus was observed (Figure 1). After the bleeding was controlled, the operation was ended. The patient was discharged a day after the operation after the vital signs were checked and an abdominal examination was performed. A week after the hysteroscopy, the pelvic examination was normal. Four months after the procedure (April 2015), the patient was readmitted to our clinic with delayed menstruation and spontaneous pregnancy was detected (last menstrual period March 12^th^, 2015). The patient experienced no problems throughout her pregnancy. On December 12^th^, 2015, a 3124 g 51 cm male infant with the Apgar score 9/10 was delivered by its head in a planned cesarean section. During the exploration, a rupture involving all layers was observed in the area of the previous rupture of the hysteroscopic myomectomy (Figure 2). It was repaired using a double-layer closure with 1.0 absorbable sutures (Figure 3).

## DISCUSSION

Hysteroscopy is a procedure that is commonly used in daily practice for diagnosis and a variety of operations. It is considered as a safe procedure with a low incidence of adverse effects. The rate of complications was reported as 0.28% among 13.600 operations in the Netherlands by Jansen et al.^(1)^ and by Aydeniz et al.^(5)^ as 0.22% among 21.676 hysteroscopies in Germany. Operative hysteroscopy is associated with perioperative and late complications^(2)^.

Uterine rupture can be observed as a perioperative complication and also late in the course of pregnancy. It is a clinically important situation because it can lead to fetal death and maternal morbidity. Uterine perforation and resection are known causes of uterine rupture. Previously reported cases of uterine rupture subsequent to hysteroscopy occurred between weeks 22 and 41. It is most likely to happen in the last weeks of pregnancy but there are reported cases of uterine rupture in the 22^nd(6)^ and 23^rd^ weeks^(7)^ following hysteroscopic metroplasty and fibroid resection, respectively. All of the patients that we have presented became pregnant in a period of less than a year after hysteroscopy. In case 2, the patient had uterine rupture in the 10^th^ week of her pregnancy. According to our literature search, this is the earliest rupture following hysteroscopic septum resection without a complication. Also, in case 3, the week of rupture was 22. Both are early ruptures compared with similar cases in the literature. The short interval between surgery and conception may have cause the early ruptures because no complications occurred in these cases.

According to Propst et al.^(8)^ ,the risks of complications vary according to the type of hysteroscopy. Myomectomy [odds ratio (OR): 7.4] and septum resection (OR: 4.0) had the highest incidence of complications, and diagnostic hysteroscopy (OR: 0.5) and polypectomy (OR: 0.1) had the lowest risks. In case 1, 2, and 3, hysteroscopic septum resection was performed, and in case 4 the hysteroscopic procedure was myomectomy. Both operations are considered as high-risk for complications. Therefore, the type of the hysteroscopy may be a predictive factor.

In case 4, the patient underwent myomectomy. During hysteroscopy, a small-sized (7-8 mm) rupture was observed, which was different from the other cases. The patient became pregnant approximately 4 months after surgery, which is a short interval even compared with the patient who had the earliest week of uterine rupture (Case 2). Although she had a previous small rupture, she had no problems during her pregnancy and the rupture was incidentally detected during exploration at cesarean section. This case leads us to two different questions: Did the rupture heal during the four-month-interval and dehiscence occur when the uterus was enlarged because of pregnancy? The second question asks whether it is possible to become pregnant despite a non-repaired rupture and not experience any complications during pregnancy?

In cases 1 and 4, the uterine rupture was detected late, in the 38^th^ week, and in labor, respectively. The patients delivered healthy babies. The interval was approximately nine months in case 1, and four months in case 4. In cases 2 and 3, the interval was 6 months and the patients experienced uterine rupture early in the course, at the 10^th^ and 22^nd^ weeks.

As a conclusion, the course of uterine rupture in pregnancy after hysteroscopic surgery may depend on many variables, such as the type of surgery, presence or absence of complications such as uterine perforation, the interval between hysteroscopy and pregnancy, and the patient’s obstetric history. Uterine rupture may present with abdominal pain and other symptoms, and also can remain silent until delivery when it is detected in exploration. It can happen as late as the last week of pregnancy and as early as the 10^th^ week of pregnancy. Physicians should inform their patients about the possible complications of hysteroscopy and warn them about becoming pregnant in a short time period after surgery because it may increase the risk.
